# Acupuncture for radicular pain: a review of analgesic mechanism

**DOI:** 10.3389/fnmol.2024.1332876

**Published:** 2024-03-26

**Authors:** Hong-Lin Li, Yi Zhang, Jian-Wei Zhou

**Affiliations:** ^1^School of Acupuncture and Tuina, Chengdu University of Traditional Chinese Medicine, Chengdu, China; ^2^Academy of Traditional Chinese Medicine Sciences, Chengdu, Sichuan, China

**Keywords:** radicular pain, acupuncture, mechanical compression, inflammation, spinal cord synapses, brain function, analgesic mechanism

## Abstract

Radicular pain, a common and complex form of neuropathic pain, presents significant challenges in treatment. Acupuncture, a therapy originating from ancient traditional Chinese medicine and widely utilized for various pain types, including radicular pain, has shown promising outcomes in the management of lumbar radicular pain, cervical radicular pain, and radicular pain due to spinal stenosis. Despite its efficacy, the exact mechanisms through which acupuncture achieves analgesia are not fully elucidated and are the subject of ongoing research. This review sheds light on the current understanding of the analgesic mechanisms of acupuncture for radicular pain, offering valuable perspectives for both clinical application and basic scientific research. Acupuncture is postulated to relieve radicular pain by several mechanisms: peripherally, it reduces muscle spasms, lessens mechanical pressure on nerve roots, and improves microcirculation; at the molecular level, it inhibits the HMGB1/RAGE and TLR4/NF-κB signaling pathways, thereby decreasing the release of pro-inflammatory cytokines; within the spinal cord, it influences synaptic plasticity; and centrally, it modulates brain function, particularly affecting the medial prefrontal cortex, anterior cingulate cortex, and thalamus within the default mode network. By acting across these diverse biological domains, acupuncture presents an effective treatment modality for radicular pain, and deepening our understanding of the underlying mechanisms regarding analgesia for radicular pain is crucial for enhancing its clinical efficacy and advancement in pain management.

## 1 Introduction

Nucleus pulposus (disk) herniation, spinal stenosis or vertebral degeneration can all lead to radicular pain and induce inflammatory processes affecting nerve roots and/or stimulation of the dorsal root ganglion, triggering dermatomal symptoms such as numbness and sharp pain (Stafford et al., 2007; Rathmell, 2008). In this paper, we provide a comprehensive review on radicular pain, including lumbar radicular pain, cervical radicular pain and spinal stenosis-related radicular pain (Patel and Perloff, 2018). The pathophysiology of radicular pain is complex, leading to less-than-optimal clinical treatment outcomes and a substantial negative impact on patients’ quality of life. Patients often prefer surgical intervention to conservative management due to its quicker relief (Ropper and Zafonte, 2015) despite comparisons 1 year after surgery showing minimal differences in symptom relief and functional improvement compared to conservative therapy alone. Therefore, surgical decompression should be recommended primarily for patients experiencing refractory or unbearable symptoms, as the long-term outcomes for radicular pain management do not significantly differ between surgical and conservative approaches (Peul et al., 2007; Lequin et al., 2013). In addition, another form of management, namely drug therapy, can lead to side effects such as nausea, fatigue and dizziness (Qaseem et al., 2017). In China, there is a tendency among patients to avoid oral analgesics for pain relief due to general dissatisfaction with the prescribed treatments (Wong et al., 2018). Moreover, Chu et al. (2023) report a unique case of a 74-year-old woman who experienced significant improvement in her L5 radiculopathy symptoms, which were initially unresponsive to traditional treatments, following chiropractic care for an L2/3 disk herniation. This case highlights the effectiveness of chiropractic rehabilitation, including spinal manipulative therapy and motorized traction at the L2/3 level, and emphasizes the need to consider non-adjacent disk herniations in radiculopathy diagnoses, offering a valuable perspective on alternative therapeutic strategies for radiculopathy. These comprehensive investigations underscore the complexity of radicular pain management, revealing that both surgical and conservative treatments offer comparable long-term outcomes while also highlighting the efficacy and patient preference for alternative therapies like chiropractic care, thereby setting the stage for the further exploration of acupuncture as another promising traditional Chinese medicine (TCM) approach in treating radicular pain.

Acupuncture has been widely used for treating various pain symptoms, including radicular pain, for thousands of years. However, standardizing acupuncture treatment presents several challenges, including the variability in acupuncture point selection, which differs among practitioners and patients (Lee et al., 2020), making uniform treatment protocols difficult to establish. The depth of needle insertion and the duration of treatment, both crucial for efficacy, also vary significantly based on the patient’s condition and body type, adding to the standardization complexity (Xu et al., 2023). Furthermore, the practitioner’s technique, which highly influences treatment outcomes, varies widely (Sherman et al., 2005). Despite these challenges, efforts toward standardization, such as the World Health Organization’s benchmarks for acupuncture practice (WHO, 2021), aim to improve treatment consistency and safety. However, the individualized nature of acupuncture, a core aspect of its philosophy, requires a balance between standardization and personalization, and ensuring safe practice is important, as adverse effects, though generally rare, might occur (Xu et al., 2023). This balance between standardizing practices for consistency and maintaining the individualized approach that defines acupuncture underscores the ongoing efforts to enhance the therapy’s effectiveness and safety.

Notably, acupuncture has demonstrated efficacy in reducing inflammatory and neuropathic pain (Mulleman et al., 2006; Zhang et al., 2014), as well as exhibiting a significant impact on the management of radicular pain, characterized by a low incidence of adverse effects (Ji et al., 2015; Qin et al., 2015) and cost-effectiveness (Kim et al., 2015). Research, including a meta-analysis, has shown that acupuncture, along with massage, provides superior outcomes compared to traction therapy and traditional Chinese medicine in the context of lumbar disk herniation treatment (Mo et al., 2019). Feng et al. (2023) reported that acupuncture was more effective in reducing pain intensity compared to sham interventions or no treatment, and adverse events related to acupuncture were mild and reversible. Furthermore, clinical evidence suggests that acupuncture delivers better short-term relief of leg pain in patients suffering from chronic discogenic sciatica than sham acupuncture (Huang et al., 2019). Another study further reported that acupuncture not only offers significant therapeutic benefits over physical exercise for lumbar intervertebral stenosis but also achieves higher patient satisfaction compared to drug therapy (Oka et al., 2018). Additional studies corroborate the efficacy of acupuncture in alleviating pain associated with elderly cervical spondylotic radiculopathy, underlining its therapeutic potential (Yang J. et al., 2023).

## 2 Acupuncture exerts analgesic effects by relieving mechanical compression of nerve roots

Compression of nerve roots is a primary cause of radicular pain. Acupuncture has been shown to alleviate this condition by relaxing muscle spasms and diminishing local edema, which, in turn, lessens the pressure on nerve roots (Fujiwara et al., 2017). Wang T. et al. (2020) reported that acupuncture at the Jiaji points could ameliorate local tissue adhesion, alleviate muscle spasms and enhance tissue oxygenation and blood flow, thereby mitigating local pain and symptoms of nerve compression. In a study focusing on cervical spondylotic radiculopathy (CSR) patients, Xu et al. (2022) observed that acupuncture could decrease the size of calcified lesions in the cervical ligament, easing nerve root compression and exerting an analgesic effect. Furthermore, Zhao et al. (2014) investigated the impact of acupuncture on rats subjected to autologous nucleus pulposus transplantation to model radicular compression and observed that acupuncture could reduce edema in the nerve roots and dorsal root ganglia, thereby improving the condition of nerve root compression.

The blood supply to nerve roots is distinct from that of other peripheral nerves, making them particularly vulnerable to mechanical injury, which significantly affects their blood circulation. Such injuries, especially due to protruding intervertebral disks, can impair arteriovenous reflux, leading to local functional ischemia, inflammatory edema and an accumulation of acid metabolites in nerve roots. These changes can result in decreased nerve root conduction function and initiate symptoms of radicular pain (Wu and Yang, 2013). Inoue et al. (2008) demonstrated acupuncture’s ability to notably reduce low back pain and lower limb pain in patients with spinal stenosis and lumbar disk herniation (LDH). They utilized a laser Doppler flowmeter to monitor changes in the blood flow of the sciatic nerve in rats, suggesting that acupuncture might temporarily modify sciatic nerve blood flow, impacting the cauda equina and nerve root circulation as a potential mechanism for its effectiveness in treating radicular pain. Furthermore, Li X. et al. (2017) indicated that combining acupuncture with traction therapy can significantly alleviate radicular pain symptoms in patients with CSR. A key mechanism behind this combined approach appears to be its capacity to enhance blood rheology parameters and promote circulation, which may subsequently reduce vascular and nerve compression, highlighting a promising avenue for treating radicular pain through acupuncture, particularly when integrated with traction methods.

## 3 Acupuncture exerts analgesic effects by anti-inflammatory action

Historically, it was widely believed that radicular pain was mainly due to mechanical compression of nerve roots; however, clinical evidence has since shown that pain severity does not always correlate with the degree of compression and showed neuroinflammation, among other mechanisms, in the pathogenesis of radicular pain (Zhao et al., 2017; Huang et al., 2018). It is now increasingly recognized that the stimulation of nerve roots by inflammatory factors significantly contributes to the development of radicular pain. Following nerve root damage, a surge in pro-inflammatory cytokines such as interleukin 1β (IL-1β), interleukin 6 (IL-6) and tumor necrosis factor-α (TNF-α) occurs, promoting inflammation in nerve roots and leading to peripheral sensitization, which increases the reactivity of injured nerves to variations in afferent signals and changes in ion channel functions, intensifying pain (von Hehn et al., 2012). Research, including studies by Pan et al. (2019) and Tian and Ma (2023), supports the efficacy of acupuncture in reducing serum levels of these inflammatory cytokines in both animal models and patients with CSR, suggesting that acupuncture mediates its anti-inflammatory and analgesic effects by observing a significant decrease in serum levels of IL-6, TNF-α and IL-1β following acupuncture treatment. Despite these insights, the precise mechanisms by which acupuncture influences neuroinflammatory pathways in radicular pain remain partially understood, necessitating further investigation.

High-mobility group box 1 (HMGB1), a pro-inflammatory mediator, is actively secreted by various immune cells like activated macrophages/monocytes and passively released by necrotic cells. Recognized as a damage-associated molecular pattern (DAMP), HMGB1 binds to the receptor for advanced glycation end-products (RAGE), a crucial pattern recognition receptor, initiating an amplification of the inflammatory response (Andersson et al., 2018). This HMGB1/RAGE interaction is pivotal in the context of radicular pain; following nucleus pulposus rupture, HMGB1 is liberated and promotes an inflammatory cascade via the RAGE receptor on cell membranes. Moreover, the HMGB1/RAGE axis can trigger intracellular signaling pathways such as the mitogen-activated protein kinases (MAPK) and activate the nuclear factor kappa-B (NF-κB) pathway, leading to the release of pro-inflammatory cytokines such as IL-1β and IL-6 by neutrophils (Hudson and Lippman, 2018). Research indicates that the HMGB1/IL-1β complex, arising from the interaction between HMGB1 and IL-1β, activates the IL-1β receptor (IL-1βR), thus amplifying the inflammatory response and exacerbating radicular pain (Bianchi, 2009; Wang J. et al., 2020). The rat autogenous nucleus pulposus grafting model, a non-compressive simulation of lumbar disk nucleus pulposus protrusion, has shown that such protrusion can lead to nerve root damage and subsequent radicular pain (Kayama et al., 1996; Lu et al., 2003). Recent studies (Su H. et al., 2023; Zhang et al., 2023) have demonstrated that this model results in significantly elevated serum levels of IL-1β, IL-6, IL-8 and NF-κB, along with increased expression of RAGE protein, HMGB1 protein and mRNA in spinal nerve trunk tissue. Remarkably, after 14 days of acupuncture treatment, there were significant reductions in the serum levels of IL-1β, IL-6, IL-8, and NF-κB, as well as in the expression of RAGE protein, HMGB1 protein and mRNA within spinal nerve trunk tissue in the treatment group compared to the control group. These results highlight the potential of acupuncture to effectively attenuate inflammatory responses by targeting the HMGB1/RAGE signaling pathway in spinal nerve trunk tissue.

Toll-like receptor 4 (TLR4) plays an essential role in initiating inflammatory responses and has been increasingly recognized for its involvement in pain modulation through the interaction between glial cells and neurons. Notably, TLR4’s expression has been confirmed in the spinal cord and the dorsal horn, highlighting its significance in the nervous system’s inflammatory pathways (Wang et al., 2019; Gao et al., 2021). Research indicates that the upregulation of TLR4 and its downstream cytokines is pivotal in transmitting pain signals in neuropathic and inflammatory pain models, primarily through inflammatory mechanisms (Tanga et al., 2005; Wadachi and Hargreaves, 2006). Activation of TLR4 can lead to the induction of NF-κB, a transcription factor that regulates the expression of pro-inflammatory genes, including IL-1β and cyclooxygenase-2 (COX-2) (Ma et al., 2020; Jin et al., 2022). In particular, COX-2 is known for promoting the production of prostaglandin E2 (PGE2), a compound that further amplifies inflammatory responses. Both IL-1β and PGE2 are recognized as significant contributors to the inflammatory process (Firlej et al., 2008; Chen et al., 2013). Studies have shown that inhibiting the TLR4/NF-κB pathway can reduce the levels of IL-1β and PGE2, thereby mitigating inflammation (Firlej et al., 2008; Chen et al., 2013). Experimental evidence from research on rats with cervical spondylotic radiculopathy reveals that acupuncture treatment can lead to a significant decrease in the expression of TLR4, NF-κB, COX-2, IL-1β and PGE2 (Fujiwara et al., 2017). These findings suggest that targeting the TLR4/NF-κB signaling pathway may be an effective strategy for acupuncture to alleviate radicular pain, underscoring its potential as a therapeutic intervention for inflammatory-mediated pain conditions.

## 4 Acupuncture exerts analgesic effects by regulating the plasticity of spinal cord synapses

The process of pain generation involves a complex interplay between peripheral inputs and the plasticity of central neurons, underscoring the significance of spinal cord synaptic plasticity in the central nervous system’s functionality. It’s established that chronic pain can stem from the increased sensitivity of peripheral nociceptors in the spinal cord, coupled with persistent synaptic plasticity, highlighting the central role of synaptic changes in pain perception (Bliss et al., 2016). Research by Yang P. et al. (2023) in a rat model has demonstrated that electroacupuncture (EA) can mitigate the spinal cord compression (SCC)-induced upregulation of synaptic proteins, including α, 1, and 2, post-synaptic density protein 95 (PSD-95), and growth-associated protein 43 (GAP-43) within the spinal cord, suggesting EA’s capability to alter synaptic ultrastructure affected by CSR, thereby influencing the synaptic structure and functional plasticity to exert analgesic effects. Moreover, a study by Su J. et al. (2023) revealed that EA effectively decreases the expression of pain-associated factor c-fos and the post-synaptic membrane protein neurontin 2 by inhibiting the expression of brain-derived neurotrophic factor (BDNF), phosphorylated tyrosine kinase B (P-TrkB), calmodulin-dependent protein kinase II (CAMKII), and phosphorylated response element binding protein (P-CREB) at spinal cord synapses. This action modulates synaptic plasticity and dampens hyperactive synaptic activity, contributing to its analgesic outcomes. These findings elucidate the mechanisms through which EA influences spinal cord synaptic plasticity, offering insights into its potential as a therapeutic intervention for chronic pain management.

## 5 Acupuncture exerts analgesic effects by adjusting the functional alterations in the brain

Research on the anterior cingulate cortex (ACC) and the insular cortex (IC) has significantly advanced our understanding of neuropathic pain management and perception. Bliss et al.’s (2016) review highlights the ACC’s critical involvement in both acute and chronic pain, evidenced through rodent studies, which underscore its role in chronic conditions. They detailed how synaptic plasticity in the ACC, particularly two types of long-term potentiation (LTP) related to NMDA and kainate receptors, contributes to the affective dimensions of pain and pain-related anxiety. Meanwhile, Coffeen et al. (2011) have shown that the rostral agranular insular cortex (RAIC) plays a vital role in modulating both inflammatory and neuropathic pain in rats, suggesting its significance in pain processing. Further, Segerdahl et al. (2015) pinpoint the dorsal posterior insula (dpIns) as crucial for pain experience in humans, with findings supported by imaging techniques and paralleled by animal studies that identify a similar region essential for nociception, thereby positioning the dpIns as a prime target for pain treatment strategies. Moreover, the ACC is known to be responsive to various pain types, including inflammatory, neuropathic and cancer pain (Xiao et al., 2021), integrating the emotional aspects of pain perception (Lee et al., 2022). Animal model research has revealed specific receptors and signaling pathways in the ACC that are involved in pain processing (Xiao et al., 2021). Similarly, the IC, particularly its posterior part, is significant in sensory processing and modulation (Alonso-Matielo et al., 2023). It is intricately linked with the ACC and other components of the pain matrix, responding to a range of painful stimuli and exhibiting altered activity patterns in neuropathic pain conditions, thus playing a role in both sensory and affective dimensions of pain. The intricate relationship between the posterior IC and the ACC, involving inhibitory projections, underscores a complex network that regulates pain’s affective components. These insights into the roles of the ACC and IC underscore their importance in neuropathic pain perception and modulation, offering promising directions for developing future pain management therapies.

Pain is a complex sensory experience influenced by sensory detection, emotional responses, and cognitive assessments, leading to widespread neural activation within the brain (Garcia-Larrea and Peyron, 2013; Wang et al., 2013). The somatosensory nervous system’s mechanoreceptors and nociceptors relay signals to the brain through the spinal dorsal horn, where they undergo further processing, and injuries or diseases affecting the somatosensory system can alter these signals, leading to pain perception (Colloca et al., 2017). Recent investigations have identified that brain regions activated during pain experiences mainly include the prefrontal cortex, ACC, left IC, thalamus, and caudate nucleus (Martucci and Mackey, 2018). Functional neuroimaging has shown that acupuncture analgesia specifically targets and measurably influences these interconnected brain areas, highlighting its role in modulating brain function to achieve pain relief (Otti and Noll-Hussong, 2012), indicating that acupuncture can alter brain activity associated with pain processing, thereby providing a neurobiological basis for its analgesic effects.

Xu et al. (2018) reported that chronic neck and shoulder pain due to cervical radiculopathy is associated with altered functional connectivity between the anterior and posterior ACC and various brain regions, indicating a widespread disruption in brain network connectivity. This condition leads to an increase in brain regions showing abnormal connectivity with the posterior ACC, suggesting a significant impact on the brain’s functional architecture. In this regard, May (2008) utilized functional magnetic resonance imaging (fMRI) to demonstrate that chronic sciatica can cause changes in several brain areas, notably within the default mode network (DMN), including the posterior cingulate cortex, precuneus, medial prefrontal cortex (mPFC) and thalamus. These alterations indicate a structural and functional remodeling of the brain in response to chronic pain (Yang and Chang, 2019). Li et al. (2012) investigated the effects of acupuncture on sciatica, noting a reduction in the visual analog scale (VAS) scores, which measure pain intensity. fMRI studies revealed that, compared to healthy controls, patients with sciatica showed decreased activity in the mPFC and anterior cingulate cortex within the DMN. Notably, acupuncture treatment was able to normalize the decreased DMN activity, suggesting that acupuncture may alleviate pain by modulating DMN activity. In another research by Li X. L. et al. (2017) on acupuncture treatment for lumbar disk herniation pain, the authors revealed its potential mechanism of action through the modulation of neuronal activities in key brain areas, including the hypothalamus, midbrain, hippocampal gyrus, amygdala, and cingulate gyrus. These findings collectively suggest that acupuncture can influence brain activity in regions critical for pain perception and processing, offering a neurobiological explanation for its analgesic effects.

## 6 Limitations and prospects

Throughout this review, we have highlighted studies demonstrating the significant outcomes associated with acupuncture treatment in regard to radicular pain. However, it is important to note that these outcomes, while indicative of acupuncture’s potential benefits, may not in themselves establish a direct causal relationship between acupuncture and pain relief. The evidence presented, largely derived from clinical observations and research findings, suggests a correlation between acupuncture intervention and improved patient outcomes, and it is possible that factors beyond acupuncture contribute to the observed effects. The methodological rigor of studies examining the efficacy of acupuncture is of paramount importance in determining the exact nature of its analgesic effects. Thus, well-designed clinical trials employing randomized controlled designs, large sample sizes and standardized outcome measures are essential to control for potential confounders such as placebo effects, patient expectations, and the influence of concurrent treatments. The complexity of pain as a subjective experience, influenced by a multitude of physical, psychological, and social factors, further complicates the ability to isolate the specific contributions of acupuncture to pain relief.

Acknowledging these considerations, we emphasize that conclusions drawn from current literature should be viewed as preliminary. They serve to inform hypotheses for future research rather than to definitively demonstrate acupuncture’s causative role in mitigating radicular pain. As such, we advocate for a cautious interpretation of the data, recognizing that while the associations are promising, they are not conclusive.

To address these methodological challenges and advance the field, future research efforts must focus on elucidating the specific mechanisms through which acupuncture may exert its effects, employing rigorous experimental designs, such as the need for further randomized controlled trials, larger and more diverse patient populations, and the application of objective measures of pain and function. Additionally, longitudinal studies examining the long-term efficacy of acupuncture and its comparison with other treatment modalities would be valuable in providing a clearer picture of its role in pain management. In light of the ongoing debate within the medical and scientific communities regarding the mechanisms and efficacy of acupuncture, it is incumbent upon researchers to continue to investigate its analgesic properties with a critical and scientific approach, which will enhance our understanding and enable us to better integrate this modality into clinical practice, ensuring that patient care is guided by evidence-based principles.

## 7 Conclusion

**FIGURE 1:**
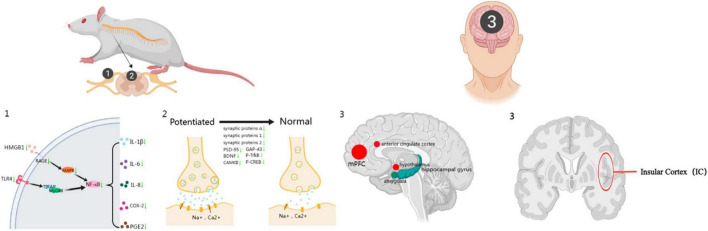
The analgesic mechanism of acupuncture. This figure illustrates the multifaceted analgesic mechanism of acupuncture. Part 1 depicts the molecular pathway involved in the inflammatory response that contributes to radicular pain. It shows the interaction of the high-mobility group box 1 (HMGB1) protein with the receptor for advanced glycation end-products (RAGE) and the subsequent activation of nuclear factor kappa-B (NF-κB) through the toll-like receptor 4 (TLR4) signaling pathway. Part 2 represents the synaptic plasticity changes in the spinal cord. It contrasts the difference between normal and potentiated synapses, highlighting the various synaptic proteins involved. Part 3 identifies key brain regions that are modulated by acupuncture in the context of pain, specifically highlighting the prefrontal cortex, anterior cingulate cortex (ACC), hippocampal gyrus, hypothalamus, amygdala, and the left insular cortex (IC) [created with permission from MedPeer (https://www.medpeer.cn)].

Although the prevalence of radicular pain is notably high, the mechanisms underlying its development have not been completely clarified. Its occurrence is often linked to factors such as compression of local nerve roots, the presence of various biochemical substances, and damage to nerve roots, alongside the transmission of pain signals (Figure 1). Acupuncture is an effective non-surgical intervention that provides substantial relief from radicular pain through actions at various anatomical levels. Peripherally, acupuncture relieves muscle spasms, diminishes mechanical pressure on nerve roots, and promotes blood microcirculation. Furthermore, it inhibits the HMGB1/RAGE and TLR4/NF-κB signaling pathways, reducing the release of pro-inflammatory cytokines and, thus, the inflammatory response, which contributes to alleviating radicular pain. Centrally, acupuncture’s analgesic properties are attributed to its influence on spinal synaptic plasticity. It also affects brain functionality, particularly impacting regions within DMN, including the mPFC, ACC and thalamus, to provide pain relief.

The role of acupuncture in treating radicular pain has been extensively validated across various disease models, including cervical spondylotic radiculopathy, lumbar disk herniation, and sciatica. Advancements in neuroscience and related fields have led to significant progress in understanding acupuncture’s mechanisms. However, there is still a notable lack of sufficient evidence to fully elucidate its enigmatic analgesic effect. Further exploration of the internal mechanisms underlying acupuncture remains imperative in the medical community. Challenges include the relatively small clinical sample sizes for acupuncture treatment of radicular pain and a lack of standardized measurement criteria. Additionally, while electroacupuncture is commonly used, further confirmation is needed to determine whether manual acupuncture yields similar regulatory effects and if differences exist compared to electrical stimulation. Incorporating supplementary results can optimize clinical operational plans. Moreover, unraveling acupuncture’s analgesic mechanism is a complex and dynamic process, yet current literature predominantly examines individual aspects without comprehensively considering their interrelationships.

Acupuncture, an important therapy in traditional Chinese medicine, plays a significant role in managing radicular pain through its diverse analgesic mechanisms. A deeper understanding of the analgesic mechanism of acupuncture is still needed for enhancing pain management strategies in clinical settings, highlighting the need for further research to elucidate the complex analgesic mechanisms associated with acupuncture.

## Author contributions

H-LL: Conceptualization, Writing – original draft, Writing – review & editing. YZ: Writing – review & editing. J-WZ: Funding acquisition, Writing – review & editing.
